# Functionalized Graphene Oxide Thin Films for Anti-tumor Drug Delivery to Melanoma Cells

**DOI:** 10.3389/fchem.2020.00184

**Published:** 2020-03-23

**Authors:** Livia E. Sima, Gabriela Chiritoiu, Irina Negut, Valentina Grumezescu, Stefana Orobeti, Cristian V. A. Munteanu, Felix Sima, Emanuel Axente

**Affiliations:** ^1^Department of Molecular Cell Biology, Institute of Biochemistry, Romanian Academy, Bucharest, Romania; ^2^Photonic Investigations Laboratory, Center for Advanced Laser Technologies, National Institute for Lasers, Plasma and Radiation Physics, Magurele, Romania; ^3^Department of Bioinformatics and Structural Biochemistry, Institute of Biochemistry, Romanian Academy, Bucharest, Romania

**Keywords:** nanomaterials, graphene oxide, thin films, MAPLE, cytotoxicity, drug delivery, melanoma

## Abstract

Since Graphene discovery, their associated derivate nanomaterials, Graphene Oxide (GO) and reduced-GO were in the forefront of continuous developments in bio-nano-technology due to unique physical-chemical properties. Although GO nano-colloids (GON) were proposed as drug release matrix for targeting cancer cells, there is still a concern regarding its cytotoxicity issues. In this study, we report on the fabrication of functional GON bio-coatings by Matrix-Assisted Pulsed Laser Evaporation (MAPLE) to be used as drug carriers for targeting melanoma cells. We first performed a thorough *in vitro* cytotoxicity assay for comparison between GON and protein functionalized GON coatings. As functionalization protein, Bovine Serum Albumin (BSA) was non-covalently conjugated to GO surface. Safe concentration windows were identified in cytotoxicity tests by live/dead staining and MTS assays for five different human melanoma cell lines as well as for non-transformed melanocytes and human dermal fibroblasts. Hybrid GON-BSA nano-scaled thin coatings incorporating Dabrafenib (DAB) and Trichostatin A (TSA) inhibitors for cells bearing BRAF^V600E^ pathway activating mutation were assembled on solid substrates by MAPLE technique. We further demonstrated the successful immobilization for each drug-containing GON-BSA assembling systems by evaluating cellular BRAF activity inhibition and histone deacetylases activity blocking, respectively. DAB activity was proven by the decreased ERK phosphorylation in primary melanoma cells (SKmel28 BRAF^V600E^ cell line), while TSA effect was evidenced by acetylated histones accumulation in cell's nuclei (SKmel23 BRAF WT cell line). In addition, melanoma cells exposed to GON-BSA coatings with compositional gradient of inhibitors evidenced a dose-dependent effect on target activity. Such functional bio-platforms could present high potential for cell-biomaterial interface engineering to be applied in personalized cancer therapy studies.

## Introduction

Carbon-based nanomaterials are extensively used in various fields of nanoscience and nanotechnology due to their exclusive physicochemical properties. Several applications are actively explored in microelectronics, energy-related materials, sensors, and biomedical field (Kuzum et al., 2014; Hong et al., 2015; Li et al., 2017). Among other low-dimensional carbon allotropes, Graphene, a two-dimensional nano-scaled carbon layer, and its derivatives, e.g., Graphene Oxide (GO) and reduced-GO exhibit distinctive advantages, such as large surface area and unique optical properties, as reviewed in Dreyer et al. (2010) and Ferrari et al. (2015). Recent advances are intensively explored in nanomedicine for the synthesis of cell-instructive microenvironments, the targeted applications being related to drug/gene delivery for cancer therapy, bacteria-killing, tissue engineering platforms, engineering stem cell responses, biosensing, and cellular imaging (Negut et al., 2018).

However, studies have shown that nanomaterials may have side-effects on health and human exposure risks were associated with both engineered and environmental nanoparticles (Chang et al., 2011; Dusinska et al., 2015; Guadagnini et al., 2015). The issue is complex since no standard reference to assess nanomaterials cytocompatibility is available. From materials point of view, there are several variables to be considered, such as nanomaterial size, shape, surface chemistry and charge, crystal structure and bandgap, dissolution, and composition-property relationship. Nevertheless, several biochemical indicators of cell proliferation, apoptosis, necrosis, oxidative stress, DNA damage could be investigated to have an integrated analysis of the biocompatibility in case of a specific foreign substance interaction with cells in culture. Predictive toxicology and high-throughput screening using compositional and combinatorial nanomaterial libraries were generally proposed to evaluate hazard scenarios (Nel et al., 2013).

It has been suggested that GO could have distinctive advantages of reduced toxicity over carbon nanotubes (CNTs), owing to its excellent aqueous solubility (without the need to use surfactant to de-bundle and disperse in water), as well as due to lack of metal catalyst impurities considered the cause of oxidative stress related to CNTs (Loh et al., 2010). It was also evidenced that the as-oxidized bare GO does not significantly interfere with A549 cell viability up to a concentration of 50 mg/L (Chang et al., 2011).

It was shown that the cytotoxicity of graphene-based nanomaterials is highly dependent on their functionalization (Oliveira et al., 2015), which gives opportunities to use this type of materials in bio-nano-technology and personalized medicine applications. Some studies evidenced that in case of 2D carbon-based nanomaterials, several parameters influences cytotoxicity (as e.g., particle size, morphology, oxygen content, surface charge, and moreover their concentration) when tested on human erythrocytes, skin fibroblasts (Liao et al., 2011) and neural phaeochromocytoma-derived PC12 cells (Zhang et al., 2010) and revealed the benefits of GO surface coating with Chitosan (Liao et al., 2011). Moreover, it has been reported that functionalized graphene, including GO and GO modified with various macromolecules such as proteins (Bovine Serum Albumine-BSA Mu et al., 2012 and Fetal Bovine Serum-FBS Hu et al., 2011), Chitosan (Liao et al., 2011), Dextran (Zhang et al., 2011), peptides (Bhunia and Jana, 2011), have significantly reduce cytotoxicity.

It became then generally accepted that the surface functionalization of graphene-based nanomaterials is critical to improve solubility, biocompatibility and availability, enhance drug-loading and release efficiency for biomedical applications. There are two main technical approaches for GO surface modification: (i) covalent functionalization, typically carried out by organic reactions, and (ii) non-covalent conjugation with molecules such as polymers (PEG), DNA, proteins, as comprehensively addressed elsewhere (Negut et al., 2018). Among several routes of surface modification, functionalization of carbon nanomaterials with proteins was found the most adequate for biomedical applications (Oliveira et al., 2015).

Matrix Assisted Pulsed Laser Evaporation (MAPLE) is an additive physical vapor deposition technique developed for functionalization of solid substrates with protein and composite coatings. When compared to other classical deposition methods (e.g., drop-cast, spin-coating, dip-coating, Langmuir-Blodgett), this method provides high experimental versatility and relatively facile control of coating thickness even for ultrathin films, the possibility to preserve materials properties even for very delicate compounds, providing adherent and uniform coatings on centimeter sized substrates, micro-fabrication of multilayers from multi-target system, and possibility to generate gradient coatings in a single-step process (Sima et al., 2016; Axente et al., 2018; Axente and Sima, 2020). However, current drawbacks are related to difficulties to fabricate large-area coatings (tens of centimeters) with high uniformity. This method allows the preparation of various drug concentrations to be immobilized as thin layers with controlled thickness covering the substrate material.

Laser-assisted synthesis of thin organic coatings of high-molecular mass such as biopolymers, proteins, and enzymes present various technical challenges. In our previous studies (Sima et al., 2016; Axente et al., 2018; Axente and Sima, 2020) we reported synthesis of highly-functional organic, inorganic and hybrid coatings, for biomedical applications. MAPLE technique proved adequate for the delicate laser transfer of biopolymers, as e.g., levan, a high molecular weight, water-soluble bacterial exopolysaccharide (β2,6-linked fructan). Levan exhibits distinct biomedical applications, having anticancer activity, anti-inflammatory, anticytotoxic, and antitumoral properties as reviewed elsewhere (Oner et al., 2016). Sima et al. (2011c) reported for the first time on pure and oxidized Levan nanostructured thin films assembling by MAPLE. The authors evidenced a high potential of cell proliferation for both coatings (with certain predominance for oxidized Levan) by *in vitro* colorimetric assays. Extracellular matrix proteins, such as fibronectin (FN) and vitronectin were also assembled as thin layers on solid substrates by MAPLE, while preserving their biological functions (Sima et al., 2011a,b). Later, our group (Sima et al., 2015), reported on the possibility to fabricate hybrid inorganic–organic thin implant coatings by laser-based techniques. Pulsed Laser Deposition was first used for the deposition of hydroxyapatite (HA) coatings, followed by MAPLE technique for assembling FN layers on top for creating a biomimetic interface for implant applications. The authors shown that <7 μg FN per cm^2^ onto HA surface is appropriate for improving adhesion, spreading, and differentiation of osteoprogenitor cells.

In this study, non-covalent surface functionalization of GO nano-colloids (GON) with Bovine Serum Albumin (BSA) protein was carried out following the protocol described elsewhere (Mu et al., 2012). We have first evaluated the cytotoxicity of GON and GON-BSA conjugates with respect to several human melanoma cell lines, as compared to normal melanocytes and human dermal fibroblasts, used as non-transformed controls. The goal is to propose a workflow for screening relevant compounds with potential anti-tumor therapeutic effect by using an innovative nano-scaled thin coating platform that contains immobilized active inhibitors for targeting key pathways and processes in cancer cells.

A laser-based approach, MAPLE, is employed herein for assembling such thin coatings on a solid substrate and fabrication of the testing platform aimed at delivery of drugs for skin cancer therapeutic response assessment. As proof-of-concept, we have incorporated BRAF and histone deacetylase (HDAC) inhibitors into GON-BSA systems and validated the functionality of these devised assemblies as molecular weapons against human melanoma cells.

## Experimental Section

### Materials

Graphene oxide nano-colloids (GON) dispersed in H_2_O (2 mg/mL), paraformaldehyde (PFA), methanol and all the reagents used for solutions were purchased from Sigma Aldrich. Bovine Serum Albumin (BSA) and goat serum were purchased from Santa Cruz Biotechnology. The signaling pathway inhibitors Dabrafenib/GSK2118436 (DAB) and Trichostatin A (TSA) were purchased from Selleckchem (www.Selleckchem.com).

### Preparation of GON-BSA Suspensions

The procedure for non-covalent surface functionalization of GON nanomaterials was performed following the protocols described in Mu et al. (2012). Briefly, GON and BSA solutions (2 mg/mL in MilliQ H_2_O) were mixed 1:1 with gentle pipetting and named GONB thereafter. After overnight (O/N) incubation at 37°C, the suspensions were centrifuged at 16 000 g for 30 min at 4°C. The pellet was then washed 3 times with PBS and centrifuged at 16 000 g, 10 min each time. Finally, GONB particles were resuspended in sterile water for further experiments. Further, six serial dilutions (3×) were performed, up to a concentration of ~1.37 μg/mL. All the solutions were UV sterilized before cell cultures experiments. Alternatively, 50 μL of each GON and GONB solutions, having fixed concentrations of 16 and 48 μg/mL, respectively were drop-casted on glass substrates of 10 × 10 mm^2^, to be tested in duplicate for each cell line.

### MAPLE Experiments

**Figure 1:**
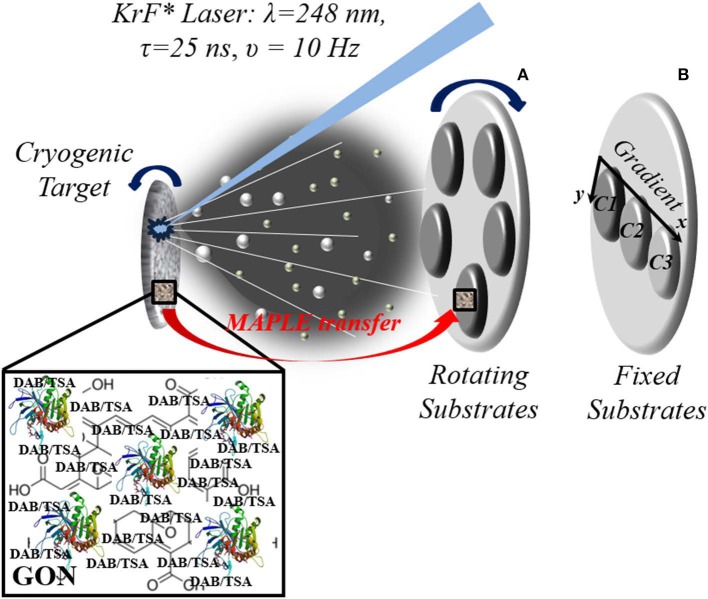
Experimental MAPLE setup exemplification for growing composite GONB-DAB/GONB-TSA coatings with fixed composition (rotating substrates) **(A)** and with compositional gradient (fixed substrates) **(B)**. Target is rotating in both configurations to avoid drilling and ensure uniform evaporation.

Detailed protocols for thin coating assembling by MAPLE were addressed elsewhere (Sima et al., 2016; Axente et al., 2018; Axente and Sima, 2020) and briefly described in the following. The procedure consists of laser irradiation of a cryogenic target by a pulsed UV laser beam (Figure 1). The target usually contains the solute molecules dissolved in an appropriate laser beam absorbing solvent. During laser irradiation, solute molecules are transferred onto a solid substrate placed parallel to the target by evaporation of the frozen solvent. Specifically, the laser-induced material ejection is generated backward from the solid cryogenic target surface. The evaporated biomolecules flux is then collected on solid substrates, growing, pulse by pulse, a thin coating of thickness from few to several hundreds of nanometers. The target was prepared by immersing aqueous solution of either GON, GONB or GONB-inhibitor in liquid nitrogen. After solidification, the target is placed inside a stainless-steel reaction chamber and evaporated using an UV excimer KrF^*^ laser source (Lambda Physics Coherent, COMPexPro 205 model) delivering pulses at λ = 248 nm, with pulse duration τ_FWHM_ = 25 ns, operated at υ = 10 Hz. Batches of thin coatings were grown on glass and silicon substrates placed parallel to the target surface at a separation distance of 40 mm. Before depositions, the substrates were cleaned in successive ultrasonic baths of acetone, ethanol, and deionized water. The substrates were kept at room temperature, the depositions being performed in a dynamic pressure of ~2 × 10^−2^ mbar. After optimization trials, 25.000 subsequent pulses of <1 J cm^−2^ incident laser fluence were applied for the synthesis of each structure. Two irradiation protocols were employed: GONB-inhibitor thin coatings with the same composition were obtained by rotating the substrates (υ_*rot*_ = 10 rot/min, Figure 1A), while coatings with compositional gradient were obtained by keeping the substrates fixed (Figure 1B). Here, the main evaporation flux was directed to sample C1, and a concentration gradient to C3 is naturally achieved due to material spreading along the radial-orthogonal direction of the substrates (*x* direction in Figure 1B). The goal of adopting this irradiation geometry was designed to generate compositional gradient coatings, in a single-step process, and to investigate a dose-dependent effect, with respect to inhibitor spatial distribution.

### Physical-Chemical Characterization of GON and GONB Nanocomposites

Morphological investigations of the nanomaterials were performed by scanning electron microscopy (SEM) (FEI-Inspect S50, Japan) and atomic force microscopy (AFM) (TT AFM Workshop, USA) in vibrating mode. All AFM images were recorded on 15 × 15 μm^2^ areas. Optical absorption spectra of the solutions were also recorded with a double beam spectrophotometer (Thermo Scientific, model Evolution 220) in the 200–900 nm wavelength range. Fourier transform infrared (FTIR) spectrometry studies were performed in absorption mode with a Shimadzu 8400S instrument. Forty scans were carried out on each sample while the investigated range was set to 500–4,000 cm^−1^ with a resolution of 4 cm^−1^ wavenumber.

### Cell Cultures

Seven model human cell lines were used in this study (see Table 1 for phenotype details). A375 and human dermal fibroblasts (HDF) were grown in DMEM high-glucose medium (Gibco, #31966), supplemented with 10% inactivated fetal bovine serum (FBS) and 1% Penicillin/Streptomycin (Pen/Strep). SKmel28, SKmel23, MelJuSo were grown in RPMI (Gibco, #61870) supplemented with 10% inactivated FBS, 1% Sodium Pyruvate, 1% HEPES, 1% Non-Essential Amino Acids (NEAA), 1% Pen/Strep. MNT-1 were grown in DMEM high-glucose medium, supplemented with 20% inactivated FBS, 10% AIM-V and 1% Pen/Strep. All supplements were from Gibco. Normal human epidermal melanocytes (NHEM) were grown in MBMTM-4 Basal Medium (#CC-3250) supplemented with MGMTM-4 SingleQuotsTM Supplements (#CC-4435) (LONZA). For experiments, the cells were counted and plated at different densities, as follows: MNT-1: 33,333/cm2, MelJuSo, Skmel28, SKmel23: 16,700/cm2, A375: 13,500/cm2, NHEM: 10,667/cm2, and HDF: 4,000/cm2 followed by culture in standard conditions or in the presence of GON/GONB colloidal suspensions or films.

**Table 1:** Cell lines characteristics.

**Cells**	**Malignancy phenotype**	**Mutation status**	**Pigmentation**
MNT-1	Primary melanoma	BRAF V600E	Highly pigmented
SKmel28	Primary melanoma	BRAF V600E	Amelanotic
MelJuSo	Primary melanoma	BRAF wt; N-Ras Q61K	Amelanotic
A375	Metastatic melanoma	BRAF V600E	Amelanotic
SKmel23	Metastatic melanoma	BRAF wt	Pigmented
NHEM	Normal primary melanocytes		Pigmented
HDF	Normal dermal fibroblasts		–

### *In vitro* Assays of Cytotoxicity and Graphene Constructs Functionality

#### Cell Viability Assessment by MTS Assay

The influence of graphene nanomaterials on melanoma cells viability and proliferation after culture in the presence of different serial dilutions of GON and GONB, respectively was assessed using CellTiter 96® Aqueous One Solution Cell Proliferation Assay kit (Promega). Cells were seeded in 96-well plates in the densities stated above and left to attach. The following day, freshly obtained GONB were added in parallel with GON as 3-fold dilution series to the cell monolayers, after 30 min UV sterilization. Cells were grown in the presence of graphene for 72 h. The optical density at 450 nm was determined 1 h after MTS reagent was added and incubated at 37°C. All the nano-colloidal solutions were tested in triplicate, against untreated cells used as controls, the results being expressed as mean values after background substraction. The absorbance values are directly proportional with the number of the metabolically active cells grown in the presence of the tested nanomaterials. For testing graphene interference with the assay, several readings were performed for comparison: (1) the 96-well plate containing the graphene dilutions and added MTS; (2) the plate containing 72 h grown A375 cells only; (1+2) the plate containing cells to which we added the graphene media dilutions plus MTS read at time t0 and after 1 h incubation at 37°C.

#### Cell Viability Assessment by Live/Dead Staining

The evaluation of cells viability and cytotoxicity of the serial dilutions of nano-colloids as well as GON and GONB thin films synthesized by MAPLE was performed using a LIVE/DEAD Viability/Cytotoxicity Assay Kit (Life Technologies) that allowed the simultaneous identification of live vs. dead cells by fluorescence assessment. The method is based on the evaluation of two characteristic parameters of cellular viability, namely the esterase intracellular activity and plasma membrane integrity. To this purpose, cells are incubated with two dyes: Calcein and Ethidium homodimer (EthD-1). The former is a non-fluorescent molecule, having the ability to penetrate by diffusion the cell membrane, which in the presence of intracellular esterase is modified to become fluorescent and is retained in viable cells. The later can penetrate the cells only if the membrane is damaged and its fluorescence is amplified up to 40 times, upon linking to nucleic acids.

We used 1 μM Calcein and 2 μM EthD-1 in PBS to stain live and dead cells, respectively, at 72 h upon seeding in the presence of graphene. The staining was performed 30 min at room temperature, followed by washing with PBS and fixing with 4% PFA to allow timely analysis of all tested samples. Finally, the samples were subjected to Hoechst nucleus staining (1:3,000) and mounting with ProLong Gold Antifade Reagent (Life Technologies). The samples were scanned with the TissueFAXSiPlus (TissueGnostics, Vienna, Austria) automated image cytometry system that allows whole specimen reconstitution for analysis and archiving.

For evaluating the cytotoxicity of the GONB/GONB-inhibitors films, cells were seeded onto the films coated glass surface and were in direct contact with the films during the course of the experiment.

#### Graphene-Inhibitors Functionality Assessment by Immunofluorescence Microscopy

All samples were investigated by fluorescence microscopy after staining with Alexa Fluor 488 Phalloidin (Life Technologies) in combination with either anti-phospho-ERK (1:1,000, Cell Signaling, #4695) or anti-acetyl histone H3 (1:1,000, Abcam, #ab47915) antibodies. After 72 h of cell culture onto GONB-DAB or GONB-TSA thin films, cells were gently washed with PBS to remove non-attached cells and serum proteins. The adherent cells were fixed with 4% PFA solution for 15 min at room temperature (RT) or with ice cold 100% methanol for 5 min at −20C°. After fixation, for phospho-ERK labeling, cells were permeabilized with 0.3% Triton-X-100 (TX) in PBS for 3 min, and then blocked for 30 min at RT in 5% goat serum diluted in permeabilization buffer. For acetyl histone H3, the cells were directly blocked in 1% BSA, 10% normal goat serum, 0.3 M glycine diluted in 0.1 % PBS-Tween for 1 h. The cells were stained with primary antibodies O/N at 4°C. After removing the excess of unbound antibodies, the cells were incubated in a buffer solution (1% BSA in 0.3% PBS-TX) containing the secondary antibody conjugated to Alexa Fluor 594 (1:400) (Life Technologies) and the Phalloidin conjugated to Alexa Fluor 488 (1:100) that binds specifically the actin filaments. After 30 min, cells were washed, stained for 1 min with Hoechst (Sigma) and then mounted on slides using the Prolong Gold Antifade Reagent solution (Life Technologies). Cells were finally analyzed using the ZEISS Axio Imager Z1 microscope and the AxioVision software. The gradient coatings were examined using the TissueFAXSiPlus system (TissueGnostics, Vienna, Austria).

## Results

### Physico-Chemical Characterization of the Nanomaterials

**Figure 2:**
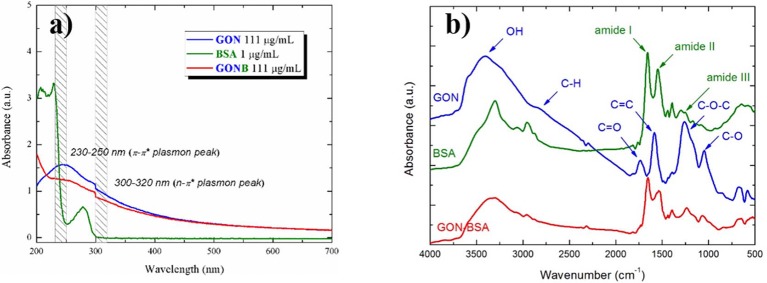
UV-Vis absorbance **(a)** and FT-IR spectra **(b)** of GON, BSA, and GONB nanomaterials.

UV-Vis absorption spectra of the starting GON, BSA and GONB solutions are presented in Figure 2a. BSA spectrum (green curve) exhibits two absorption peaks at about 220 and 280 nm. The strong one at around 220 nm reflected the absorption of the backbone of BSA, while the weak absorption peak at around 280 nm is specific to the aromatic amino acids (Trp, Tyr, and Phe) (Xu et al., 2013). In case of GON (blue curve), two plasmonic peaks in the 230–250 nm (π-π^*^) and 300–320 nm (n-π^*^) regions are attributed to C=C and C=O transitions, respectively (Konios et al., 2014). GONB spectrum (red curve) contains both GON and BSA peaks. This represents the evidence of the GON-BSA mixture and the first confirmation of the successful GON functionalization with BSA protein.

The UV-Vis results were supported by the IR absorption spectra, with respect to successful non-covalent GO conjugation with BSA. In Figure 2b there are shown the spectra of GON (blue curve), BSA (green curve), and GONB (red curve) in the 4,000–500 cm^−1^ range. GON spectra is characterized by the absorption bands of –OH (~3,400 cm^−1^), C=O (~1,700 cm^−1^), C=C (~1,600 cm^−1^), C-O-C (~1,200 cm^−1^) and C-O (~1,050 cm^−1^) vibrations (Konios et al., 2014). Protein spectra is dominated by the vibration bands characteristic to amides (I, II, and III) at around 1,650, 1,540, and 1,250 cm^−1^, respectively (Pauthe et al., 2002). GON functionalization with BSA is evidenced in the mixed GONB spectra that contains all the peaks belonging to the initial materials.

Next, we used the functionalized GONB nanomaterials to include two different signaling pathway inhibitors with potential use as anti-melanoma drugs: dabrafenib (DAB) and Trichostatin A (TSA). In order to validate the functionality of our proposed assemblies we performed experiments to estimate the drug loading content and the released fractions of TSA from GONB nanoparticles in different pH conditions. In order to eliminate possible sample handling artifacts, we opted for the analysis of the unbound and released TSA fractions by direct infusion using nanoESI (ElectroSpray Ionization) and High-Resolution Mass Spectrometry (HRMS). Previous works have shown that direct injection MS analysis of small molecules can be used to estimate their concentration level in a sample (Zhang et al., 2004; Alfazema et al., 2005). We first tested the linearity of the signal by infusing solutions with different concentrations of standard TSA (Supplementary Figure 1I). We observed a linear signal for concentrations up to low μM, confirming previous results (Zhang et al., 2004; Alfazema et al., 2005). We next interrogated unbound and released samples, for TSA identification and quantification. Analysis of the precursor ions confirmed the presence of the TSA protonated precursor molecular ion at m/z 303.17 in both categories of samples (Supplementary Figures 1C–E). The m/z was similar to the one obtained by simulation of the TSA spectrum (Supplementary Figure 1A) and with the ion identified in the standard sample (Supplementary Figure 1B). Further comparison of the MS/MS fragmentation spectra with the standard (Supplementary Figure 1F) confirmed the identification of TSA in both unbound and released samples (Supplementary Figures 1G,H). We defined quality criteria for the confirmation of TSA in each analyzed sample as described under *Supplementary Materials and Methods* section. Once confirmed (Supplementary Table 1), we next estimated by regression analysis the level of TSA based on the precursor ion intensity measured in each sample (Supplementary Figure 1J). We assessed the TSA concentration in the unbound fraction in the lower nanomolar, corresponding to <5% of TSA remained compared with the starting concentration of 125 μM (Supplementary Figure 1I, red dot corresponds to the estimated TSA). Furthermore, our results revealed a decreasing pattern, with time, of the TSA concentration level in the released samples, regardless of the pH used for sample collection (Supplementary Figure 1J).

### Morphological Investigations of the Thin Nanomaterial Films

**Figure 3:**
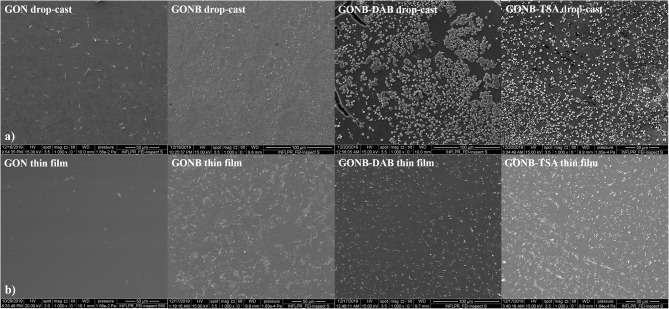
SEM images of drop-casted **(a)** and thin coatings **(b)** for GON, GONB, GONB-DAB, and GONB-TSA deposited on Silicon substrates.

**Figure 4:**
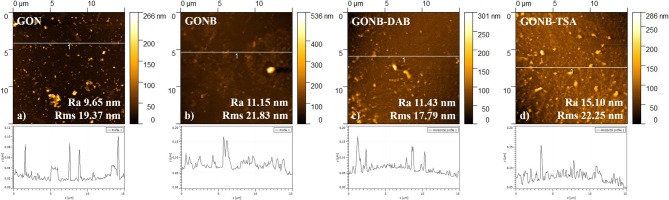
AFM images and height profiles of GON **(a)**, GONB **(b)**, GONB-DAB **(c)**, and GONB-TSA **(d)** thin films grown on Si, after scanning of 15 × 15 μm^2^.

Figure 3 shows SEM images of GON, GONB, GONB-DAB, and GONB-TSA drop-casted (Figure 3a) and thin coating grown on Silicon substrates by MAPLE (Figure 3b). The thin coatings look uniform, compact and do not present any sign of cracks and peeling, evidencing a high adherence to the substrates. Smoother coating surfaces could be evidenced in case of GON thin films while the presence of the protein was found to change drastically the morphology, which results in higher roughness. Micrometers-sized regular structures could be related to the crystallized salts from the buffer solutions transferred along with the protein on solid substrates (Sima et al., 2011b). Nanoscale features of the coatings were evaluated by AFM topographical analyses on the surfaces. Figure 4 presents 2D images of GON, GONB, GONB-DAB, and GONB-TSA thin coatings along with their surface roughness parameters and height profiles. In Figure 4a, the spatial distribution of a single graphene sheet of 1 μm lateral dimension and 20 nm height can be noticed from GON sample. In comparison, GONB revealed a significant morphological modification with surface height profiles changing accordingly (Figure 4b). In addition, the roughness parameters of the GONB surfaces (Ra and Rms) were found higher compared to those obtained on simple GON (Figure 4b). GONB-DAB thin coatings exhibited similar topographical characteristics with GONB samples (Figure 4c), whereas GONB-TSA indicated the highest roughness values (Figure 4d). More insights in the surface topographical aspects can be found in Supplementary Figure 2 where the 3D surface profiles are depicted.

### Cytotoxicity Assays of Nanocomposite Solutions and Thin Films

#### Quantitative Assessment of Cytotoxic Potential of Graphene Colloidal Solutions

In order to assess the cytocompatibility degree of both GON/GONB solutions and thin films, we have performed cell biology experiments on representative cell lines, on both normal phenotype cells e.g., dermal fibroblasts and melanocytes (used as normality controls) and cell lines exhibiting various stages of tumorigenicity encountered in malignant melanoma (A375, MJS, MNT-1, SKmel23, SKmel28).

The goal of the first set of experiments was to identify the maximum graphene nanoparticles concentrations that can be safely applied without inducing cytotoxic effects, to be further assembled in form of thin coatings by MAPLE. In addition, we compared the simple and functionalized nanomaterials in order to identify the best host matrix for melanoma inhibitor embedding and release. An ideal delivery platform should not affect the normal skin cells, while the tumor cells should undergo apoptosis following exposure to targeted drugs with inhibitory activity against specific signaling pathways that mediate tumors growth and resistance to chemotherapeutics.

**Figure 5:**
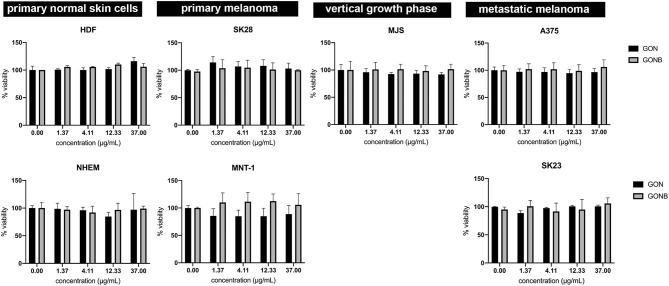
MTS assay results showing no cytotoxicity for both GON (black columns) and GONB (gray columns) on controls and malignant skin cells up to concentrations of 37 μg/mL.

**Figure 6:**
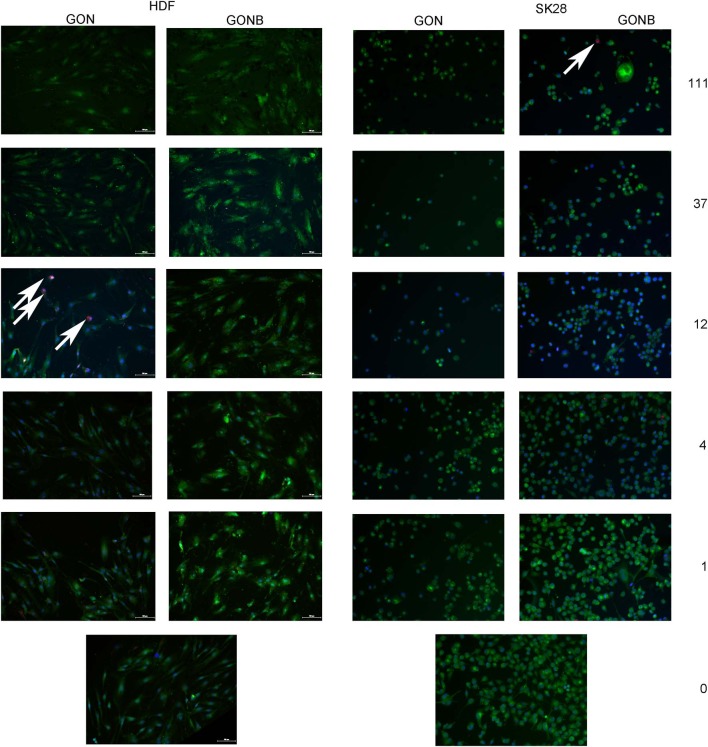
Representative fields of view obtained by TissueFAXS scanning of GON and GONB solutions treated HDF or SK28 cells, evidencing superior biocompatibility of BSA functionalized GON (GONB) when compared to simple GON. The white arrows indicate the EthD-1 stained dead cells nuclei. Viable cells stained with calcein are visible in green. Scale bar = 100 μm.

We have first investigated the influence of GON and GONB 3-fold serial dilutions (in the range of 0–1,000 μg/mL) on both primary control cells and melanoma cell lines by quantifying metabolically active cells using the MTS assay at 72 h upon treatment. During the optimization process we observed that graphene preparations interfere with absorbance measurements at 450 nm. Using A375 melanoma cells we tested the contribution of adding GON or GONB nanoparticle suspensions onto cells in the presence of MTS reagent before and after 1 h incubation. Results showed that there is no interference from GOs on OD measurements at 450 nm up to 37 μg/mL for GON or 111 μg/mL in case of GONB (Supplementary Figure 3). Therefore, we sought to test the cytotoxic potential of GOs when added in suspension to normal skin cells or melanoma using concentrations up to 37 μg/mL. The viability data as function of nanomaterial concentrations are presented in Figure 5. Results showed no significant viability decrease at concentrations up to 37 μg/mL for both GON and GONB on all melanoma cell lines tested in this study, representative for primary, vertical growth phase, and metastatic cancer, as well as for normal skin cells (Figure 5). In order to confirm the results, we next performed a live/dead double staining of HDF and SKmel28 cells. Immunofluorescence microscopy results have shown that GONB are not inducing any toxic reaction in HDF at concentrations up to 111 μg/mL while simple GON evidenced cytotoxicity signs at 12 μg/mL (Figure 6, HDF). Analyses of melanoma cells showed ~ 100% viable cells in all tested conditions (Figure 6, SK28). In order to confirm assay specificity, we evaluated both calcein and EthD-1 optimized concentrations on untreated cells or cells treated for 10 min with ethanol. As shown in Supplementary Figure 4, dermal fibroblast cells (Supplementary Figures 4A,B) and melanocytes (Supplementary Figures 4C,D) grown in standard conditions (a and c) are fully viable, while all those fixed in ethanol (b and d) exhibit red-emitting nuclei.

By correlating the live/dead staining (Figure 6) and MTS assays (Figure 5), we were able to identify safe concentration windows for both nanomaterials.

#### Testing the Cytotoxic Potential of Nanomaterial MAPLE Thin Films

Prior to laser deposition experiments, we tested the viability of HDF cells when grown onto GON and GONB drop-casts obtained from solutions at two different concentrations: 16 and 48 μg/mL, respectively. Cell viability was preserved in all tested conditions (Supplementary Figure 5); plain glass substrate was used as positive control whereas ethanol treatment was used as negative.

**Figure 7:**
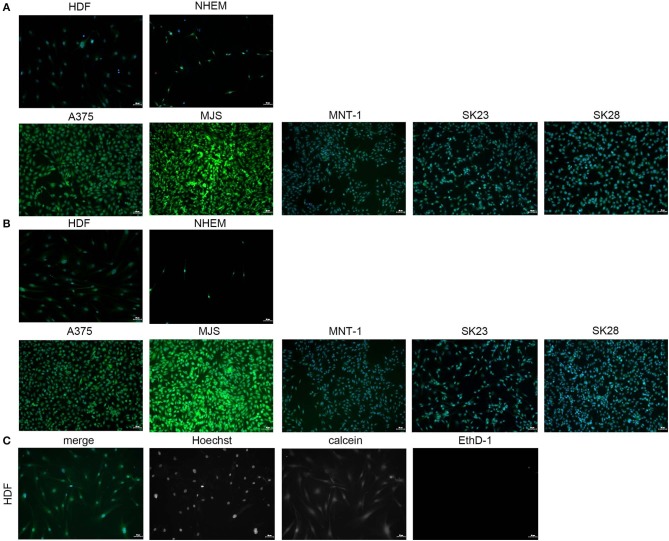
Immunofluorescence microscopy images obtained by scanning GON **(A)** and GONB **(B)** thin films obtained by MAPLE upon culture with normal skin cells and melanoma cells. The images revealed high biocompatibility for both GON and GONB thin films on control and human melanoma skin cells grown after 72 h. Viable cells are shown in green due to calcein staining. Dead cells were not detected. Merged images were obtained by overlapping the TissueFAXS captured fields of view on each fluorescence channel for detection of Hoechst, calcein, and EthD-1 **(C)**. Images correspond to HDFs grown on standard cover slip borosilicate glass. Scale bar = 50 μm.

Thin coatings were obtained by MAPLE technique starting from GON and GONB cryogenic targets (see coating surface morphologies and topographies in Figures 3, 4). Similar to testing of nanomaterials in colloidal suspension, and as drop-cast, calcein/EthD-1 double staining was performed after 72 h culture, followed by immunofluorescence microscopy analyses to assess GON (Figure 7A) and GONB (Figure 7B) cytotoxic potential when presented to cells as deposited thin coatings. Figure 7C shows the obtaining of the merged images by overlapping the captured fields of view generated on each fluorescence channel, upon fixation and Hoechst nuclei labeling. After scanning the entire samples, no dead cells were detected for all MAPLE samples, with respect to whole spectrum of cell lines used in this study (representative fields of view in Figure 7). This complements the MTS assay results obtained on nanomaterial solutions (Figure 5). The images revealed biocompatibility for both GON and GONB thin films on normal primary melanocytes and dermal fibroblasts, as well as human melanoma cells grown for 72 h onto MAPLE graphene coatings.

### Investigation of Functionalized GONB MAPLE Thin Films Embedding Inhibitors

**Figure 8:**
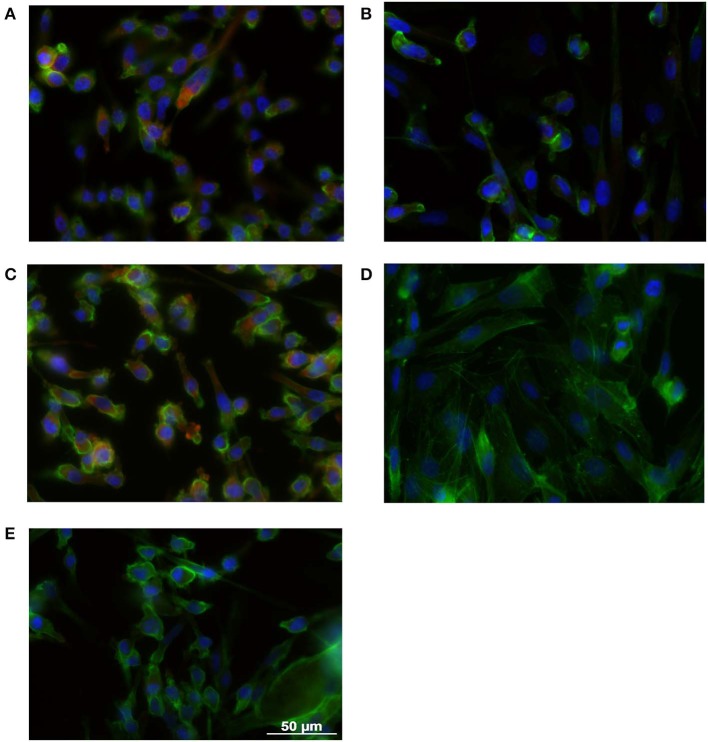
Immunofluorescence microscopy images obtained on SK28 human melanoma cell line grown on GONB MAPLE thin films without **(A)** or with **(B)** DAB BRAF inhibitor. SK28 malignant cells grown in standard conditions in the absence **(C)**, or in the presence **(D)** of 0.5 nM DAB were used as controls of DAB activity. The comparison with SK23 melanoma cells that express wild type BRAF is presented in **(E)**. Cells were stained with anti-phospho-ERK antibodies (red) and Phalloidin (green) for exhibiting MAPK pathway activation and the cellular cytoskeleton consisting of actin filaments, respectively. Nuclei were stained with Hoechst (blue). Scale bar = 50 μm.

Benefiting from the fact that GONB thin films do not induce cytotoxic effects *per se*, we have addressed the possibility to fabricate functional thin films incorporating inhibitors for targeting specific signaling pathways/processes associated with malignant transformation. First, we have chosen a BRAF inhibitor, Dabrafenib (DAB), a drug already approved by the FDA in 2013 for the treatment of advanced metastatic melanoma bearing the mutated BRAF gene (BRAF^V600E^) found in ~70% of melanoma tumors (Davies et al., 2002). It has the advantage of a low IC_50_ index, which makes it effective at nM concentrations. Thin coatings containing DAB in the GONB matrix were assembled on glass substrates by MAPLE. The resulting nano-layers (thickness <50 nm) were tested against SKmel28 cell line, which exhibits the mutant version of the BRAF gene, that constitutively activate the MAPK signaling pathway downstream of this kinase. As a direct effect, a continuous phosphorylation of ERK protein is observed (Wan et al., 2004). The clear proof of DAB activity is quantified in BRAF inhibition and corresponds to a decreased phosphorylated ERK (pERK) expression. In order to evaluate inhibitor potency upon embedding in the MAPLE deposited graphene layers, we performed immunofluorescence microscopy using specific antibodies against pERK in combination with phalloidin that allows visualization of the entire cell based on the actin cytoskeleton footprint (Figure 8). Indeed, one could easily notice that the pERK protein expressed in BRAF^V600E^ SKmel28 cells grown on GONB coated glass (Figure 8A), is not found in cells grown up to 72 h on DAB containing GONB thin films (Figure 8B). As control conditions for these findings, SKmel28 melanoma cells were grown on standard cover slips in the absence (Figure 8C) or presence of 0.5 nM DAB (Figure 8D), a situation resulting in a similar total suppression of pERK expression. SKmel23 cell line that contains the unmodified version of BRAF gene and which consequently express low steady-state levels of pERK protein were used as negative technical controls for pERK staining specificity (Figure 8E).

**Figure 9:**
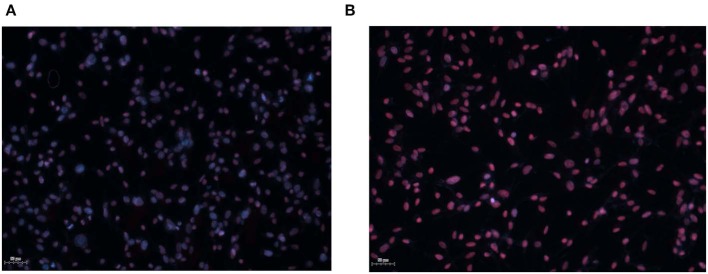
Immunofluorescence microscopy images obtained on SK23 human melanoma cell line grown on GONB MAPLE thin films without **(A)** or with **(B)** TSA HDAC inhibitor. Cells were stained with anti-acetyl histone H3 antibodies (red) and Hoechst (blue) for nuclear labeling. Scale bar = 50 μm.

Second, we have tested the functionality of a histone deacetylase (HDAC) inhibitor, Trichostatin A (TSA), an epigenetic modulator that is currently tested in clinical trials for relapsed or refractory hematologic malignancies. Recently, HDAC inhibitors were shown to dramatically enhance the efficacy of BRAF/MEK inhibitors in sensitive and insensitive RAS pathway–driven melanomas (Maertens et al., 2019). A direct effect of HDACi treatment is the accumulation of acetylated histones in the nucleus, the substrates of histone deacetylases. Acetylated histones are known as epigenetic marks of active genes (Roh et al., 2005). Use of HDACi is expected to recover tumor suppressor genes activity that was lost during the cancer transformation process (Li and Seto, 2016). To validate TSA inhibitor activity upon embedding in the GONB matrix and laser deposited coatings, we performed immunofluorescence microscopy to assess accumulation of acetyl histone H3 in the nucleus of melanoma cells (Figure 9). Our results show that culture of SKmel23 melanoma cells for 72 h on TSA containing GONB films induces increase in nuclear signal corresponding to anti-acetyl histone labeling (Figure 9).

**Figure 10:**
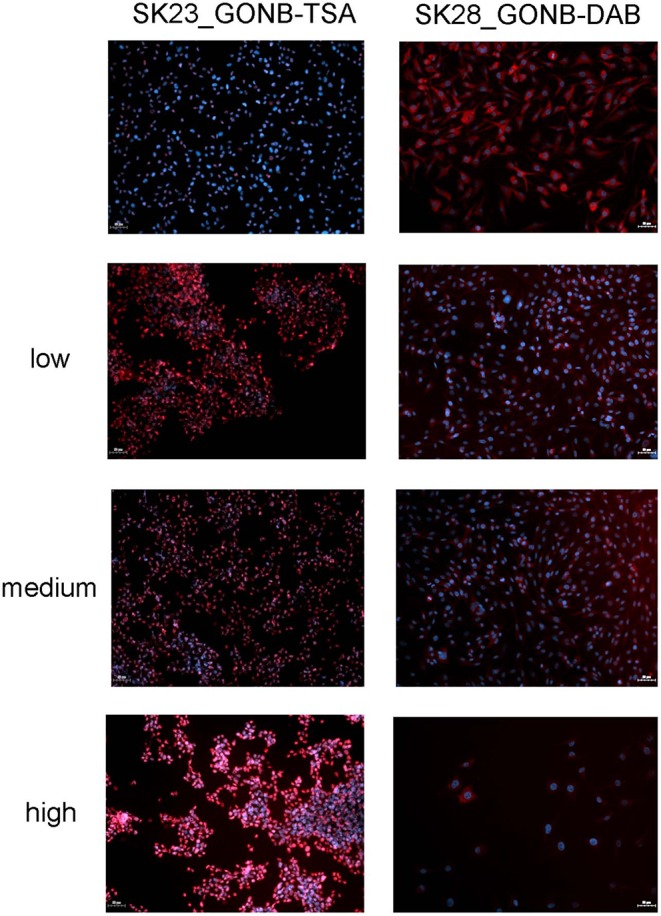
GONB-DAB and GONB-TSA gradients (from low to high) functionality was tested by immunofluorescence microscopy as in Figures 8, 9, respectively, in comparison with GONB only substrates. Expressions of pERK and acetylated histone H3, respectively are labeled in red while nuclei are labeled in blue (Hoechst). Scale bar = 50 μm.

With a view to demonstrate the high potential for development as drug combinations screening platform, our next aim was to validate the MAPLE grown GON-BSA coatings with compositional gradient of inhibitors by exposing the melanoma cells to its content and explore the potential dose-dependent effect. For this purpose, we used the already described immunofluorescence approaches to assess the dose-dependent response of cells to different concentrations of graphene-inhibitor complexes. Increasing concentrations of GONB-TSA induced an increase in fluorescence signal intensity and proportion of SKmel23 cells expressing acetyl histone H3 in the nucleus (Figure 10, left panel). Also, increasing concentrations of GONB-DAB induced a stepwise decrease in pERK signal of SKmel28 cells (Figure 10, right panel) as compared to cells treated with GONB alone (Figure 10, right panel, top image). Simultaneously, we observed a decrease in cell density on graphene coatings carrying a higher concentration of inhibitors, which motivates us to conduct more in-depth studies in the future in order to determine the conditions ensuring specific targeting of cancer cells.

Our results have demonstrated functionality of DAB and TSA inhibitors after laser immobilization in GONB matrix and subsequent assembly as thin coating, against specific molecular targets in melanoma cells. All these results are proving the high potential of functionalized GON nanoscale thin coatings to uptake different signaling pathway inhibitors without losing their activity.

## Discussion

In this study, we demonstrate the congruent and controlled transfer by MAPLE of functional assembling systems of GON containing BSA and anti-cancer drugs, on solid substrates. BSA is a neutral protein with a high molecular weight of about 66.5 kDa, having various biochemical applications. It was used with this study for GON functionalization to minimize potential graphene cytotoxicity and as a drug-carrier. Accordingly, one would expect that laser-induced damage of BSA, would result in inhibitors damage and/or loss of functionality. Noteworthy, during the laser mediated process, the drug molecules preserved their inhibitory activities when included in the GONB matrices. Dabrafenib is an inhibitor that proved beneficial activity in clinical trials of phase 1 and 2 in patients with BRAF^V600E^-mutated metastatic melanoma (Huang et al., 2013). Its chemical formula: C_23_H_20_F_3_N_5_O_2_S_2_, reveals a complex stoichiometry and the presence of several volatile elements that could be irreversibly damaged by laser irradiation, and thus, the severe alteration of inhibitor properties. TSA has the chemical formula C_17_H_22_N_2_O_3_, exhibiting a complex molecular structure, being an HDAC epigenetic inhibitor used in phase 1 of clinical trials. We demonstrated that GONB-DAB and GONB-TSA composite coatings are effective against melanoma cells during *in vitro* assays. Our choice of compounds for this proof-of-concept study was based on the high therapeutic potential of BRAF and HDAC inhibitors. BRAF inhibitors are targeting the most prevalent melanoma-driving mutation, BRAF^V600E^ (Davies et al., 2002) and several molecules in this category are FDA approved (NIH U. S. National Library of Medicine, 2019). HDAC inhibitors are a promising class of compounds that showed low side effects while constituting an alternative treatment for hematological as well as solid malignancies (Johnstone, 2002; Kelly and Marks, 2005). Two HDAC inhibitors (Vorinostat and FK228) are already FDA approved for the treatment of cutaneous T-cell lymphoma.

MAPLE method preserved drug inhibitor activity of DAB and TSA, as proven by the specific effects on their intracellular molecular targets. Decrease of ERK phosphorylation with increasing gradient concentration of DAB was obtained. Also, nuclear accumulation of acetylated histones in melanoma cells exposed to GONB-TSA was recorded.

Future Combinatorial MAPLE (C-MAPLE) produced thin film bio-platforms (Oner et al., 2016; Axente and Sima, 2020), incorporating other drugs or active substances could be efficient for testing drug combinations co-targeting a specific cancer cell population. Indeed, once safe concentration windows on nanomaterials cytotoxicity are identified, further functionalization offers the possibility to create smart materials, exhibiting improved properties for melanoma therapeutic screening. The main mechanism associated with BRAF inhibitors resistance is the reactivation of the MAPK pathway (Alcala and Flaherty, 2012; Manzano et al., 2016). It is critically important to understand the mechanisms of resistance to targeted therapy and find the means to overcome this situation to maximize patient's survival. In 2017, FDA approved DAB administration in combination with Trametinib, another MAPK inhibitor, for the treatment of a subtype of patients with non-small cell lung cancer bearing the BRAF^V600E^ mutation. These premises extend the possibility to use combinations including DAB also in other cancers (Ji et al., 2012). HDAC inhibitors were shown to sensitize melanoma cells to BRAF/MEK inhibitors (Maertens et al., 2019). These examples of therapeutic combinations could be further extended using efficient miniaturized platforms able to support the high throughput micro-screening of compounds on biopsies or circulating tumor cells (Tolcher et al., 2018).

Our current research efforts are dedicated to laser-based fabrication of thin coatings with multiple drug combinations for testing their synergy and anti-tumor efficacy.

## Conclusions

BSA and anti-cancer drug functionalized GON nanomaterials were successfully immobilized on solid substrate platforms by MAPLE technique and tested with melanoma cells. GON and GONB treatment determined no significant cell viability decrease in normal and melanoma cells up to concentrations of 37 μg/mL in water solutions. When exposed to MAPLE deposited films, cells showed optimal viability on films obtained from targets containing up to 12 μg/mL for GON and up to 111 μg/mL for GONB when testing on human melanoma and normal fibroblast cells. These safe concentration windows were considered for starting solutions in laser experiments for deposition of inhibitors-containing GONB thin coatings. Hybrid GONB thin coatings incorporating DAB and TSA inhibitors, respectively, were further assembled on solid substrates by MAPLE. It was demonstrated that they preserve drug activity against cells bearing BRAF^V600E^ activating mutation. TSA embedded GONB thin coatings were shown to maintain histone deacetylase inhibitor activity proved by evident accumulation of acetylated histones in treated cancer cells. Thus, the successful laser immobilization of anticancer drugs within GON-BSA matrix was demonstrated by their efficient activities for BRAF and HDAC inhibition. This type of functional bio-platforms present high potential as miniaturized high-throughput platforms for drug screening, and for testing cancer cell response to different drugs and drug doses in precision medicine applications.

## Data Availability Statement

The raw data supporting the conclusions of this article will be made available by the authors, without undue reservation, to any qualified researcher.

## Author Contributions

EA and LS coordinated the project, organized the experiments and tasks, supervised all the experiments, analyzed data, wrote, edited, and reviewed the manuscript. IN and VG performed MAPLE experiments and physical-chemical investigations. LS and GC designed and realized *in vitro* assays and analyzed the data. SO performed AFM and contributed to the realization of *in vitro* assays. GC performed inhibitors release assay. CM performed mass spectrometry analyses. FS supervised MAPLE experiments and physical-chemical characterizations and reviewed the manuscript.

### Conflict of Interest

The authors declare that the research was conducted in the absence of any commercial or financial relationships that could be construed as a potential conflict of interest.
