# Supplementary material for: Functionalized Graphene Oxide Thin Films for Anti-tumor Drug Delivery to Melanoma Cells
Source: Front Chem. 2020 Mar 23;8:184. doi: 10.3389/fchem.2020.00184 (PMC7104690; doi:10.3389/fchem.2020.00184)
Supplement: Supplementary file 1 [file Presentation_1.pdf]

Supplementary Material

Functionalized Graphene Oxide Thin Films for Anti-tumor Drug Delivery to Melanoma Cells

Livia E. Sima [1*], Gabriela Chiritoiu [1], Irina Negut [2], Valentina Grumezescu [2], Stefana Orobeti [1,2], Cristian V.A. Munteanu[3], Felix Sima [2], and Emanuel Axente [2*]

[1]Department of Molecular Cell Biology, Institute of Biochemistry, Romanian Academy, 296 Splaiul Independentei, 060031, Bucharest, Romania

[2]Photonic Investigations Laboratory, Center for Advanced Laser Technologies, National Institute for Lasers, Plasma and Radiation Physics, 409 Atomistilor Street, 77125, Magurele, Romania

[3]Department of Bioinformatics and Structural Biochemistry, Institute of Biochemistry of the Romanian Academy, 296 Splaiul Independenței 060031, Bucharest, Romania

1. Supplementary Data

Materials

All LC/MS grade chemicals (formic acid, acetonitrile, ammonium acetate, ammonium bicarbonate) were purchased from Sigma Aldrich. Borosilicate emitters were from Thermo Scientific.

Methods

Sample preparation for mass spectrometry analysis

TSA release from GONB nanoparticles was performed by mixing in an 1:1 (V:V) ratio the nanoparticles with distinct buffers covering a wide range of pH scale: solvent A (0.1% formic acid - FA + 2% acetonitrile - ACN) for pH~3 (acidic), 25 mM ammonium acetate for pH~6 (close to neutral) and 25 mM ammonium bicarbonate for pH~9 (alkaline). The release was performed at 37 ^oC with slowly agitation for various times: 3, 24 and 48h. Samples were centrifuged at 16000 *x g* for 30 min and the supernatant from each sample was further processed for mass spectrometry analysis, alongside the unbound sample.

High-Resolution Mass Spectrometry (HRMS) analysis

Before injection the samples were diluted in solvent A and ~ 5 µl of the solution were further infused for HRMS analysis. All the experiments were performed on a high-resolution instrument LTQ-Orbitrap Velos Pro mass spectrometer (Thermo Fisher Scientific, Bremen, Germany). The samples were

analyzed by direct injection using metal-coated borosilicate emitters (Thermo Scientific) interfaced with the instrument by the Nanospray offline kit (Thermo Scientific) with the Nanospray Flex ion source. For sample analysis a custom method was used, which involved the acquisition of positive SIM scans in the Orbitrap at the 100 000 resolution (m/z 400) for several minutes, in order to detect the ions corresponding to the precursor molecular ion. All the samples were analyzed by infusion at a spray voltage of 1.2-1.4 kV, a capillary temperature of 275 °C and S-lens RF level of 60%, which allowed a stable spray for analysis. For MS/MS analysis, precursors were isolated in the linear trap (LTQ) and subject to CID (Collision Induced Dissociation) with helium at normalized collision energy of 35 eV and an activation q value of 0.225. Fragments ions were further sent to C-trap accumulation for Orbitrap detection.

HRMS data analysis

For data exploration the XCalibur v2.1 (Thermo Fisher) software package was used. The analysis involved validation of TSA identification in each sample and estimation of the signal intensity of the precursor molecular ion. For a positive identification the maximum allowed mass accuracy of the precursor and fragment ions was 20 ppm and validation also involved the assignment of the precursor and at least three fragment ions also observed in the MS/MS fragmentation of the standard. Quantification was performed by integrating the ion current corresponding to the protonated molecular precursor for ~ one minute.

2. Supplementary Figures and Tables

2.1 Supplementary Figures

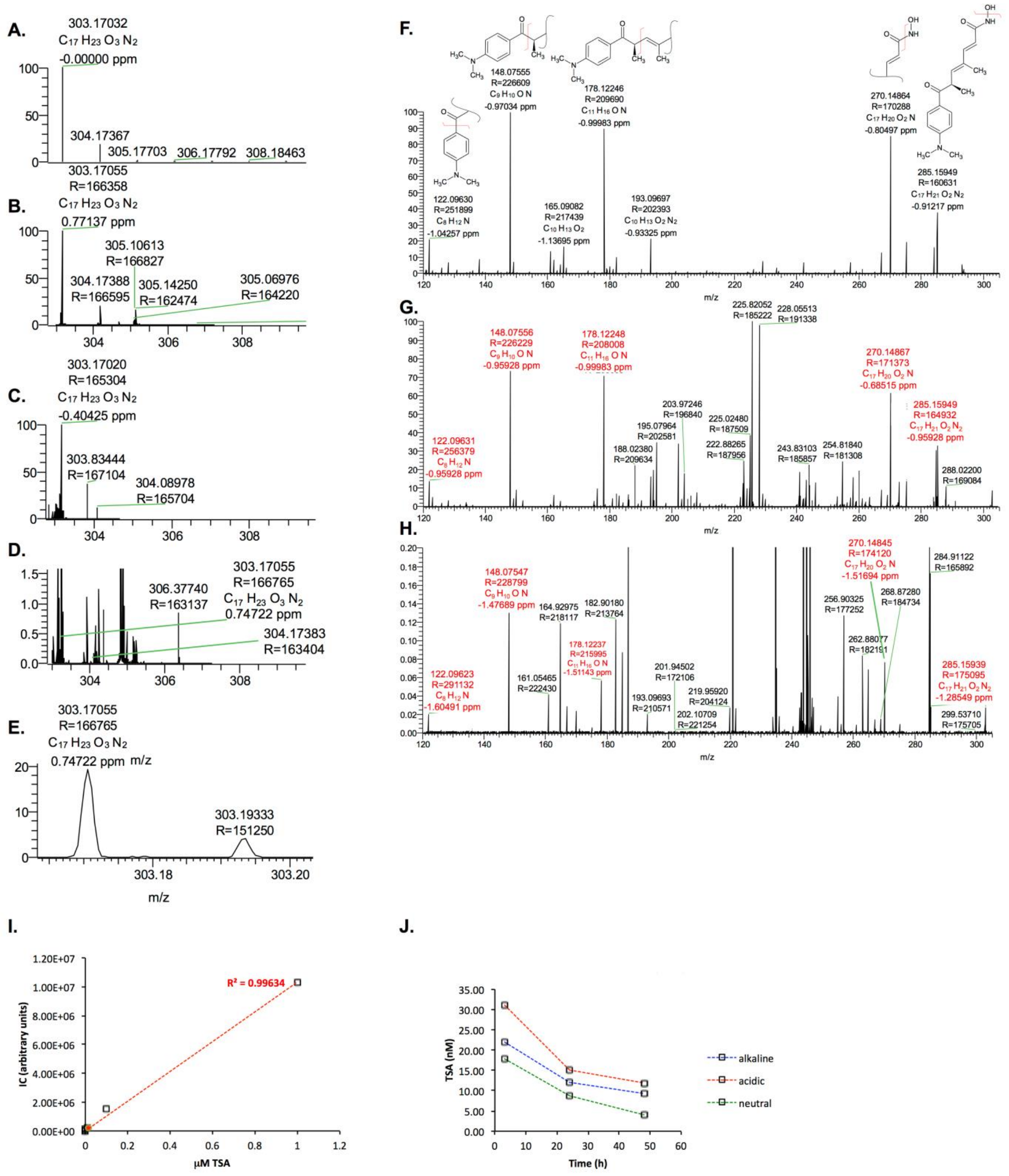

Supplementary Figure 1. nano-ESI HRMS analysis of TSA. A-E. Protonated precursor molecular ion pattern in the simulated spectrum **(A)**, standard sample **(B)**, unbound sample **(C)** and released **(D)**. **E.** Zoom-in of the spectrum in D, evidencing the precursor ion. For each set the measured m/z, the resolution, the molecular formula and the mass accuracy are denoted. It can be observed mass accuracies in the low ppm. **F-H.** CID MS/MS fragmentation spectra of TSA identified in the standard sample **(F)**, unbound **(G)** and released sample **(H)**. For each spectrum are shown the experimental m/z, the resolution, the molecular formula and the proposed structures and the mass accuracies. For the unbound and released samples the TSA main fragment ions are evidenced in red. **I.** TSA calibration curve reveals signal linearity up to the low µM range. **J.** TSA estimated concentration across different time points and pH.

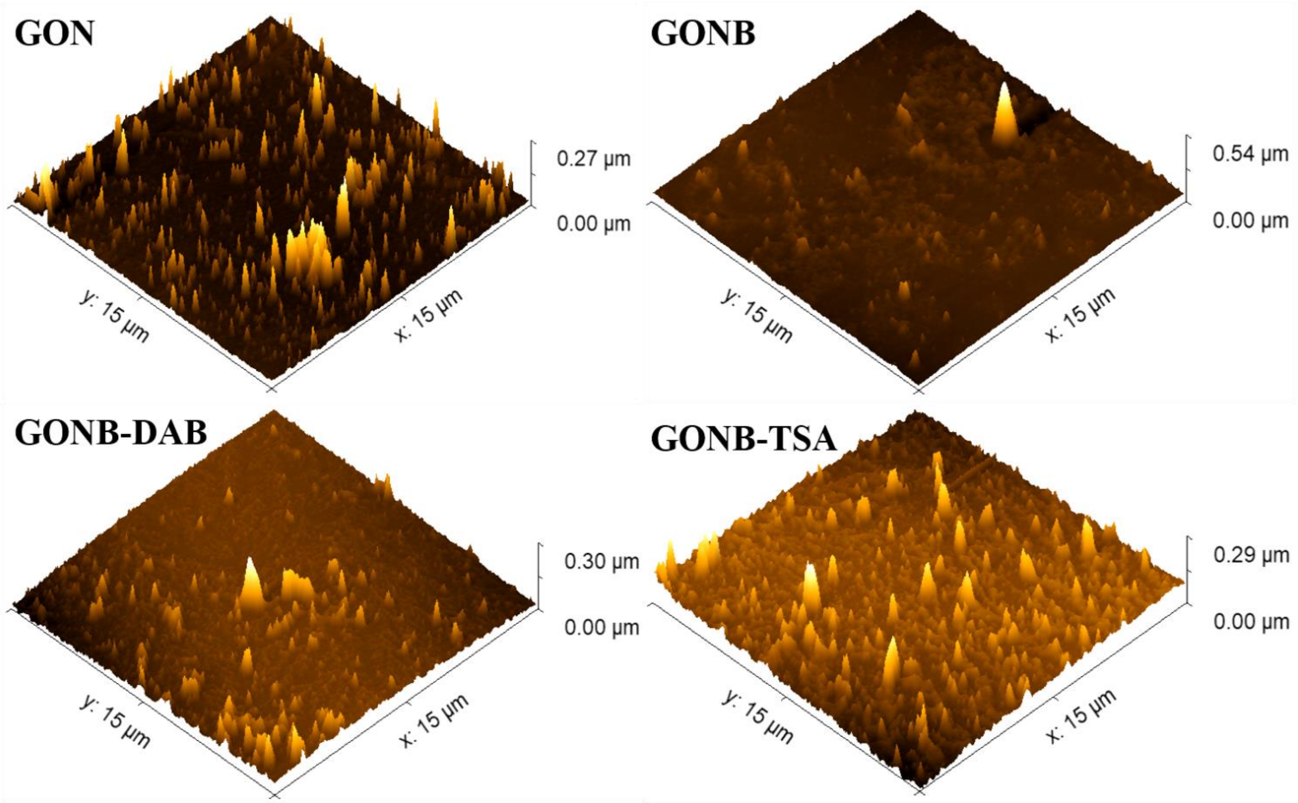

Supplementary Figure 2. 3D AFM images of GON, GONB, GONB-DAB and GONB-TSA thin coatings grown on Si, after scanning of 15×15 μm^2.

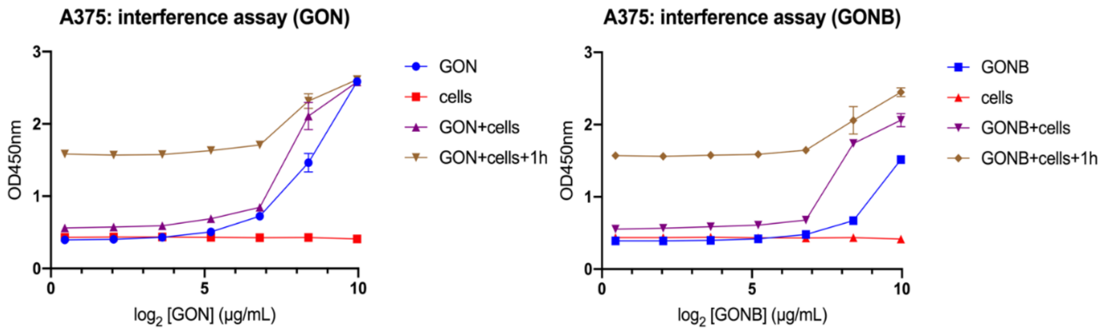

Supplementary Figure 3. Interference test of graphene nanoparticles with MTS assay performance. A 3-fold serial dilution of GON (left) or GONB (right) was prepared. The absorbance of graphene (blue) was compared to that of graphene added to A375 melanoma cells before (purple), and after incubation for 1h in the presence of MTS reagent (brown). Untreated cells were considered as baseline (red). GON starts interfering with measurements of MTS assay at 37 µg/mL while GONB interfered with the assay starting at 111 µg/mL.

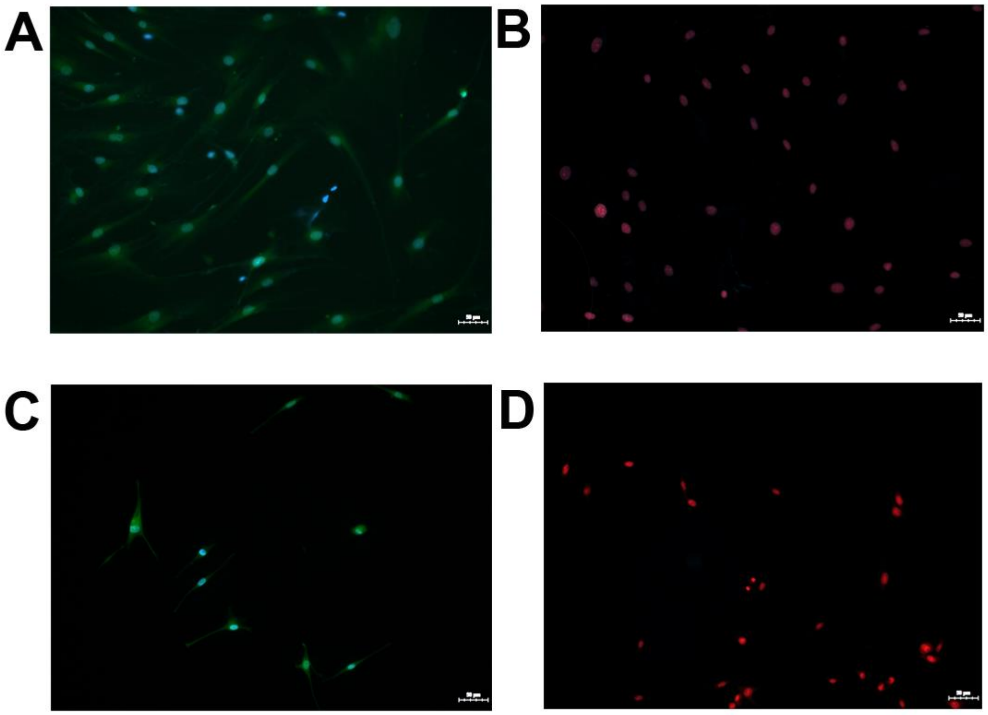

Supplementary Figure 4. Immunofluorescence microscopy images showing calcein and EthD-1 staining specificity of viable cells (**A,C**) and dead cells after ethanol treatment (**B,D**), respectively. The tested cells are representatives for normal cell lines in human skin – primary dermal fibroblasts (HDF-**A,B**) and primary melanocytes (NHEM-**C,D**). Scale bar = 50 µm.

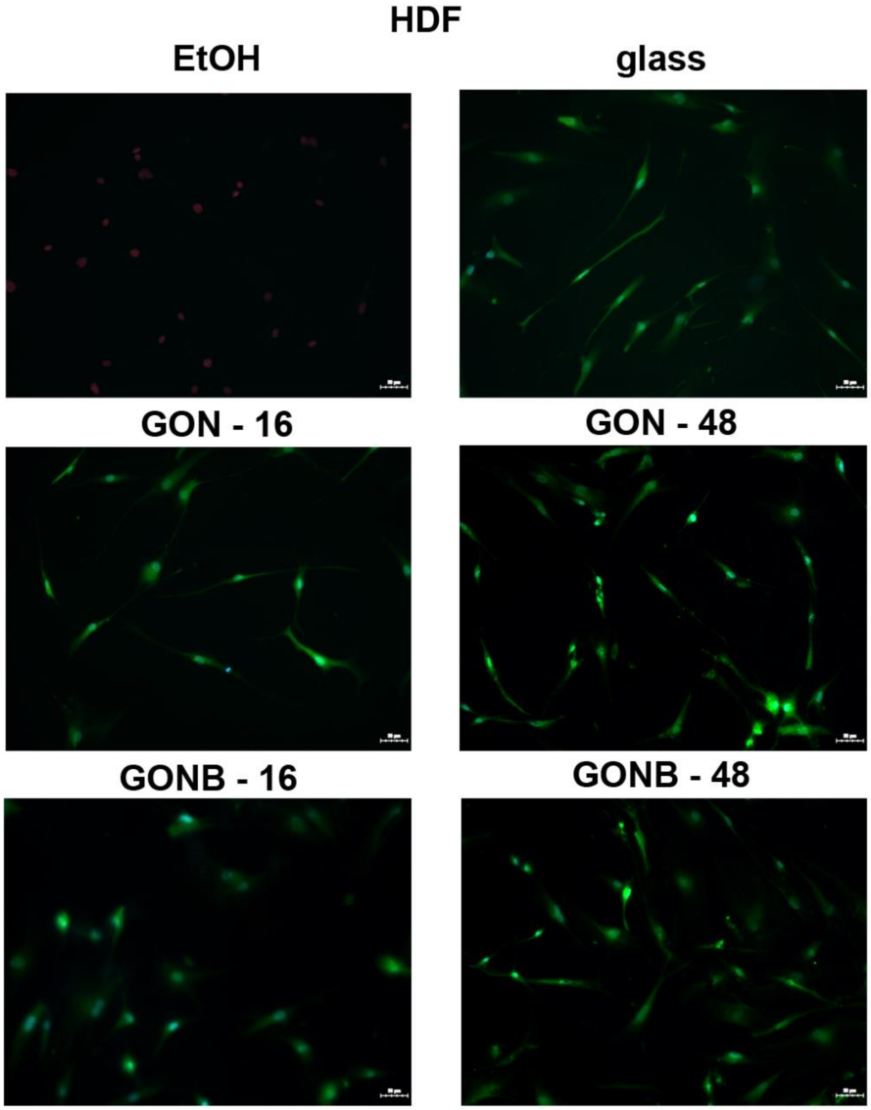

Supplementary Figure 5. Live/dead assay of GON and GONB dropcast cytotoxicity on HDF cells. Plain glass was used as control substrate while ethanol was used as positive staining control for EthD-1. Two concentrations of graphene were used to generate the dropcasts: 16 and 48 µg/mL, respectively. There was no evident toxicity in HDF cultures in the conditions tested. Scale bar = 50 µm.

2.2 Supplementary Table

Supplementary Table 1. Assignment of TSA identification in the analyzed released samples. Shown are the proposed molecular formula, the experimental m/z, mass accuracy and the sample collection conditions.

Formula	m/z exp	Mass accuracy (ppm)	Molecular ion	pH	Time (h)
$C_{17}H_{23}O_3N_2$	303.17042	0.34666	$[M+H]^+$	acidic	3
$C_8H_{12}N$	122.09622	-1.64832	fragment		
$C_9H_{10}ON$	148.07545	-1.59233	fragment		
$C_{11}H_{16}ON$	178.12237	-1.53737	fragment		
$C_{17}H_{20}O_2N$	270.14837	-1.79253	fragment		
$C_{17}H_{21}O_2N_2$	285.15934	-1.4374	fragment		
$C_{17}H_{23}O_3N_2$	303.1705	0.59725	$[M+H]^+$		24
$C_8H_{12}N$	NA	NA	fragment		
$C_9H_{10}ON$	148.07549	-1.33348	fragment		
$C_{11}H_{16}ON$	178.12239	-1.40671	fragment		
$C_{17}H_{20}O_2N$	270.14847	-1.41063	fragment		
$C_{17}H_{21}O_2N_2$	NA	NA	fragment		
$C_{17}H_{23}O_3N_2$	303.17044	0.40667	$[M+H]^+$		48
$C_8H_{12}N$	NA	NA	fragment		
$C_9H_{10}ON$	148.07541	-1.91528	fragment		
$C_{11}H_{16}ON$	NA	NA	fragment		
$C_{17}H_{20}O_2N$	270.14829	-2.08558	fragment		
$C_{17}H_{21}O_2N_2$	285.15471	-17.68143	fragment		
$C_{17}H_{23}O_3N_2$	303.17056	0.80873	$[M+H]^+$	neutral	3
$C_8H_{12}N$	122.09623	-1.60491	fragment		
$C_9H_{10}ON$	148.07547	-1.47689	fragment		
$C_{11}H_{16}ON$	178.12237	-1.51143	fragment		
$C_{17}H_{20}O_2N$	270.14845	-1.51694	fragment		
$C_{17}H_{21}O_2N_2$	285.15939	-1.28549	fragment		
$C_{17}H_{23}O_3N_2$	303.17055	0.77787	$[M+H]^+$		24
$C_8H_{12}N$	NA	NA	fragment		
$C_9H_{10}ON$	148.07534	-2.37785	fragment		
$C_{11}H_{16}ON$	178.12218	-2.57813	fragment		
$C_{17}H_{20}O_2N$	270.1482	-2.43833	fragment		
$C_{17}H_{21}O_2N_2$	285.15905	-2.478	fragment		
$C_{17}H_{23}O_3N_2$	303.17058	0.87624	$[M+H]^+$		48
$C_8H_{12}N$	122.09621	-1.76106	fragment		
$C_9H_{10}ON$	148.07544	-1.72367	fragment		
$C_{11}H_{16}ON$	NA	NA	fragment		
$C_{17}H_{20}O_2N$	NA	NA	fragment		
$C_{17}H_{21}O_2N_2$	285.1594	-1.25757	fragment		
$C_{17}H_{23}O_3N_2$	303.17043	0.36367	$[M+H]^+$	alkaline	3
$C_8H_{12}N$	NA	NA	fragment		
$C_9H_{10}ON$	148.07545	-1.65676	fragment		
$C_{11}H_{16}ON$	178.12232	-1.81167	fragment		
$C_{17}H_{20}O_2N$	270.1484	-1.6827	fragment		
$C_{17}H_{21}O_2N_2$	285.15929	-1.62245	fragment		
$C_{17}H_{23}O_3N_2$	303.1706	0.92531	$[M+H]^+$		24
$C_8H_{12}N$	122.09624	-1.53055	fragment		
$C_9H_{10}ON$	148.07548	-1.43175	fragment		
$C_{11}H_{16}ON$	178.12241	-1.32178	fragment		
$C_{17}H_{20}O_2N$	270.14856	-1.07649	fragment		
$C_{17}H_{21}O_2N_2$	285.15943	-1.12138	fragment		
$C_{17}H_{23}O_3N_2$	303.17059	0.881	$[M+H]^+$		48
$C_8H_{12}N$	122.09626	-1.31883	fragment		
$C_9H_{10}ON$	148.0755	-1.31007	fragment		
$C_{11}H_{16}ON$	178.12238	-1.47539	fragment		
$C_{17}H_{20}O_2N$	270.15307	15.60369	fragment		
$C_{17}H_{21}O_2N_2$	285.15932	-1.50596	fragment		
